# Transcriptome analysis and characterization of genes associated to leaf tannin content in foxtail millet [*Setaria italica* (L.) P. Beauv.]

**DOI:** 10.1186/s12864-022-08746-8

**Published:** 2022-07-14

**Authors:** Suying Li, Yanjiao Cui, Dan Liu, Zilong Zhao, Jing Zhang, Zhengli Liu

**Affiliations:** 1 Department of Life Sciences, Tangshan Normal University, Tangshan, China; 2 Tianjin Key Laboratory of Crop Genetics and Breeding, Institute of Crop Sciences, Tianjin Academy of Agricultural Sciences, Tianjin, China

**Keywords:** Foxtail millet, Chinese chestnut, Red spider, Tannin, Transcriptome analysis, Differentially expressed genes

## Abstract

**Background:**

Chinese chestnut is an economically important tree species whose yield and quality are seriously affected by red spider attack. Tannins is one of the most important class secondary metabolites in plants, and is closely associated with plant defense mechanisms against insect and herbivory. In our previous studies, it was revealed that several low-tannin foxtail millet varieties growing under the Chinese chestnut trees could attract red spiders to feed on their leaves and protect the chestnut trees from the infestation of red spiders, meanwhile, the growth and yield of foxtail millet plants themselves were not greatly affected.

**Results:**

To identify genes related to leaf tannin content and selection of foxtail millet germplasm resources with low tannin content for interplanting with Chinese chestnut and preventing the red spider attack, the leaves of 4 varieties with different levels of tannin content were harvested for comparative transcriptome analysis. In total, 335 differentially expressed genes (DEGs) were identified. For acquisition of gene functions and biological pathways they involved in, gene ontology (GO) and Kyoto encyclopedia of genes and genomes (KEGG) enrichment analyses were performed, and several DEGs were found to possibly participate in the tannins biosynthesis pathway and transport processes of precursors. In addition, according to the PlantTFDB database, some transcription factors were predicted among the DEGs, suggesting their role in regulation of tannins biosynthesis pathway.

**Conclusion:**

Our results provide valuable gene resources for understanding the biosynthesis and regulation mechanisms of tannins in foxtail millet, and pave the way for speeding up the breeding of low-tannin varieties through marker-assisted selection, which could be utilized for interplanting with Chinese chestnut trees to confer protection against red spider attack.

**Supplementary Information:**

The online version contains supplementary material available at 10.1186/s12864-022-08746-8.

## Introduction

Chinese chestnut (*Castanea mollssina* Blume) is an economically important tree species which originated from China. In Yanshan Mountain and Taihang Mountain area of Beijing–Tianjin–Hebei region, the major producing areas of Chinese chestnut in China, the planting area covered by Chinese chestnut has reached about 313.33 thousand hm^2^, and Chinese chestnut plays an irreplaceable role in economic development of this area. However, it was found that the yield and the quality of Chinese chestnut kernel decrease with the incidence of red spider (*Tetranychus cinnbarinus*), an agricultural insect pest which can damage more than 110 kinds of plants [1], thereby seriously affecting the income of local farmers. Thus, there is urgent need to find an effective approach to control the occurrence of red spiders on Chinese chestnut trees.

Insecticides have been applied to fight against red spiders, however, chemical pesticide residues pose hazards to both humans and the environment. Besides, trapping and killing red spiders, and release of natural enemies of red spiders were also effective measures, which significantly reduce the harm of red spiders to Chinese chestnut [2, 3]. Nevertheless, these biological control methods are time consuming and lead high economic cost. Therefore, it is important to explore a new way to prevent the red spider attack on Chinese chestnut, which takes into consideration the economic, social and environmental aspects.

Plant phenols have been regarded as one of the most common and widespread group of defensive compounds against insects in various studies [4–6]. Plant phenols range from simple phenol (MW 94) to polyphenols, such as anthocyanin pigments (MW 2000) and tannins (MW up to 20,000) [7]. Tannins have been reported to have strong deleterious effects on phytophagous insects affecting both growth and development by protein binding, reducing nutrient absorption efficiency and causing midgut lesions [8–10]. Mohamed and Abd-El Hameed found that the tannin content in leaves and seeds of faba bean (*Vicia faba* L.) plants is significantly increased in the insect-resistant genotypes as than in the susceptible genotypes [11], and Rizwan et al. reported that whitefly populations exhibit a significant and negative response to the leaf tannin content of cotton varieties [12], revealing the role of tannins in plant defense against insect damage.

Foxtail millet [*Setaria italica* (L.) P. Beauv.] is one of the most important commercial crops in Beijing–Tianjin–Hebei region of China, and has a strong resistance to drought and barren soil [13]. In our previous study on stereo interplanting system of foxtail millet with Chinese chestnut tree, we breed several early maturing and shade-tolerant foxtail millet materials, which could grow well under the spreading Chinese chestnut tree, realizing the high-efficient utilization of land resources under the trees. Meanwhile, we found that these materials could attract red spiders to feed on the leaves and protect the trees from the infestation of red spiders, while the growth and yield of foxtail millet plants themselves were not greatly affected. Moreover, the leaf tannin content of foxtail millet is about 10 times lower than the content of Chinese chestnut, which is probably one of the major reasons leading to attraction of foxtail millet plants for red spiders, according to the negative correlation of tannin content with insect infestation mentioned above [11, 12]. For this reason, identification of foxtail millet germplasm resources with low tannin content and using them for interplanting with Chinese chestnut may be an effective method to prevent the red spider attack on Chinese chestnut tree.

Molecular marker that tightly linked to desired genes is a powerful tool to screen genotypes of interest, which saves time and resources, and has been used in several plant species for selection of genotypes with low tannin content or zero tannin content. For example, two complementary recessive genes, *zt-1* ant *zt-2*, control the absence of tannins in faba bean, and development of tannin-free cultivars will enhance the nutritional quality of this legume [14]. Gutierrez et al. developed Random Amplified Polymorphic DNA (RAPD) based Sequence-Characterised Amplified Region (SCAR) markers closely linked to *zt-1* and *zt-2* genes and validated their effectiveness and applicability in discriminating the genotypes differing in tannin content [15, 16]. Webb et al. mined a Single Nucleotide Polymorphism (SNP) marker for *ZT1* [17]. Hou et al. reported Simple Sequence Repeat (SSR) markers for *zt1* gene, among which a marker predicted the *zt-1* genotypes with absolute accuracy [18]. Recently, Zanotto et al. reported a robust Kompetitive Allele-Specific PCR (KASP) marker associated with *zt2* gene [19]. However, no potential candidate genes related to tannin content and molecular markers were reported in foxtail millet so far. Moreover, elucidation of the molecular mechanisms underlying tannins biosynthesis also needs identification and functional analysis of key genes.

In this study, to explore genes related to tannin content in foxtail millet, we compared the transcriptomes of foxtail millet varieties with different levels of tannin content. Through examination of differential gene expression, several potential candidate genes contributed to tannins biosynthesis in foxtail millet were identified, and their GO and KEGG enrichment analyses were performed. Moreover, the transcription factors involved in the regulation of tannins biosynthesis pathway were predicted by using the PlantTFDB database. Our results provide valuable gene resources for development of molecular markers and speeding up the breeding of low-tannin varieties, and will provide a basis for understanding the biological pathways and molecular regulation mechanisms behind tannins biosynthesis in foxtail millet.

## Methods

### Plant materials

Five foxtail millet hybrid varieties, 56229, 57295, 12950, 1121, HK950, and a conventional variety, JG32, which were preserved by Tangshan Normal University, were used in this study. All these materials were planted in Beiguan Village of Qianxi County, Tangshan, China. Three standard plants were selected for each variety, and middle part of the first, second and third leaves from the top of each plant at booting stage were sampled. Then the collected leaves of 3 standard plants for each variety were mixed and ground for tannin content determination.

### Determination of tannin content

The chemical analysis to determine tannin content was carried out at ZKGX Research Institute of Chemical Technology (Plant Lab) of Chinese Institute of Chemical (Beijing, China). Tannin was determined by spectrophotometric method according to the standard NY/T 1600–2008 [20]. Briefly, the reaction mixture contained tannin extract solution, water, Folin-Denis reagent (containing Na_2_WO_4_·2H_2_O, H_3_Mo_12_O_40_P·XH_2_O and phosphomolybdic acid solution) and Na_2_CO_3_ solution was incubated at room temperature for 2 h. The absorbance was measured immediately at 765 nm using a spectrophotometer. Gallic acid was used for standard curve.

The production practice showed that the ability of foxtail millet hybrid HK950 to attract red spiders to feed was strong, while the ability of JG32 was weak. Therefore, the average (0.25) of leaf tannin content of these two varieties was used as a control to screen varieties with high or low leaf tannin content. According to the tannin content listed in Table S1, varieties 57295, 56229 and 12950 were considered as varieties with high tannin content, and 1121 was identified as a variety with low tannin content. These 4 varieties were then used for high-throughput sequencing.

### RNA extraction, cDNA library construction and Illumina sequencing

To mine genes related to leaf tannin content, the middle part of the first, second and third leaves from the top of plants at booting stage were sampled from the 4 foxtail millet hybrid varieties, 57295, 56229, 12950 and 1121. Leaves collected from 3 standard plants for each variety were mixed and frozen in liquid nitrogen for total RNA isolation. Three biological replicates were performed for each sample.

Total RNA isolation, cDNA library construction and RNA-seq were performed by Berry Genomics Co., Ltd (Beijing, China). A total of 2 µg RNA with high quality per sample was adopted to construct cDNA libraries using VAHTS mRNA-seq v2 Library Prep Kit for Illumina following manufacturer’s recommendations and index codes were added to attribute sequences to each sample. Briefly, mRNA was purified from total RNA using poly-T oligo-attached magnetic beads. Fragmentation was carried out using fragmentation buffer. First strand cDNA was synthesized and second strand cDNA synthesis was subsequently performed. Remaining overhangs were converted into blunt ends. After adenylation of 3’ ends of DNA fragments, adaptor with hairpin loop structure were ligated. Then the PCR was performed. At last, Qubit HS quantification, Agilent 2100 Bioanalyzer/Fragment Analyzer 5300 quality control, the final library size of about 350 bp. The libraries were sequenced on an Illumina NovaSeq platform to generate 150 bp paired-end reads (raw data) according to the manufacturer’s instructions.

### Assembly of RNA-seq data

The raw data (raw reads) of fastq format were firstly processed through primary quality control. In this step, clean data (clean reads) were obtained by removing read pairs that contain N more than 3 or the proportion of base with quality value below 5 is more than 20%, in any end, or adapter sequence was founded. All the downstream analyses were based on the clean data with high quality.

Paired-end clean reads were aligned to the *Setaria italica* reference genome (Setaria_italica_v2.0, http://plants.ensembl.org/Setaria_italica/Info/Index) using HISAT2 version 2.0.6 [21] and featureCounts version 2.0.0 [22] was used to count the reads numbers mapped to each gene.

### Differential expression genes analysis

The gene expression levels were determined as fragments per kilobase of transcript per million mapped reads (FPKM) value. The software edgeR version 3.3.3 [23] was used to detect the differential expression of genes between foxtail millet varieties with high leaf tannin content and low tannin content, and |log_2_(fold change)|> 1 and adjusted p-value (padj) < 0.05 were set as the threshold in the differentially expressed genes (DEGs) screening process.

### Chromosome location

Chromosome positional data including the chromosome number and position of DEGs were retrieved from Ensembl Plants (http://plants.ensembl.org/index.html). The genes were then plotted individually onto the respective foxtail millet chromosomes and the resultant physical maps were displayed using MapChart software [24].

### Quantitative real-time PCR (qRT-PCR) verification

Several DEGs were selected to verify the expression levels of RNA-Seq by qRT-PCR. The total RNA extraction, cDNA synthesis and qRT-PCR analysis were performed as described previously [25]. The sequences of primers applied in qRT-PCR were listed in Table S2. The PCR thermal profile was under the following cycle conditions: an initial 95 °C for 15 s, followed by 40 cycles at 95 °C for 10 s, and 60 °C for 31 s. All experiments were used in triplicates for each sample and the relative expression levels of each gene were calculated using 2^−ΔΔCT^ method [26].

### Gene Ontology (GO) and Kyoto Encyclopedia of Genes and Genomes (KEGG) enrichment analyses

The GO (http://www.geneontology.org) and KEGG (https://www.kegg.jp/) pathway enrichment analyses of DEGs were carried out using topGO [27] and KOBAS 3.0 [28] with default parameters, respectively.

### Transcription factor identification

The transcription factor families were identified using the Plant Transcription Factor Database PlantTFDB 5.0 (http://planttfdb.gao-lab.org/prediction.php) [29].

## Results

### Identification of DEGs

To identify possible candidate genes involved in tannins biosynthesis, estimation of tannin content was carried out among 23 foxtail millet varieties, and varieties 57295, 56229 and 12950 were selected as varieties with high tannin content, while 1121 was considered as a variety with low tannin content (Table S1). Illumina sequencing was performed on leaves of these 4 samples, with 3 replicates on each sample (Table S3 and Figure S1), and a total of 30,141 genes were acquired. According to their FPKM value in different samples, the DEGs were identified.

**Fig. 1:**
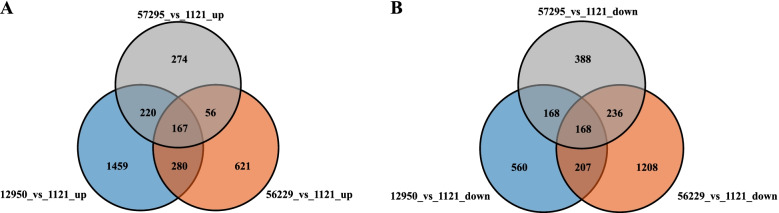
Venn diagram depicting the number of differentially expressed genes (DEGs) between foxtail millet varieties with high and low leaf tannin content. (**A**) and (**B**) shows the distribution of up-regulated and down-regulated DEGs in each comparison, respectively

In total, when compared with variety 1121, 717, 1124 and 2126 up-regulated DEGs were identified in 57295, 56229 and 12950, respectively (Fig. 1A). Among them, 167 DEGs were shared in 3 foxtail millet varieties with high tannin content. Meanwhile, there were 960, 1819 and 1103 down-regulated DEGs in 57295, 56229 and 12950, respectively, among which 168 DEGs were in common (Fig. 1B). The differential expression analysis provided amounts of potential candidate genes with respected to tannins biosynthesis in foxtail millet.

### Chromosome location of DEGs

**Fig. 2:**
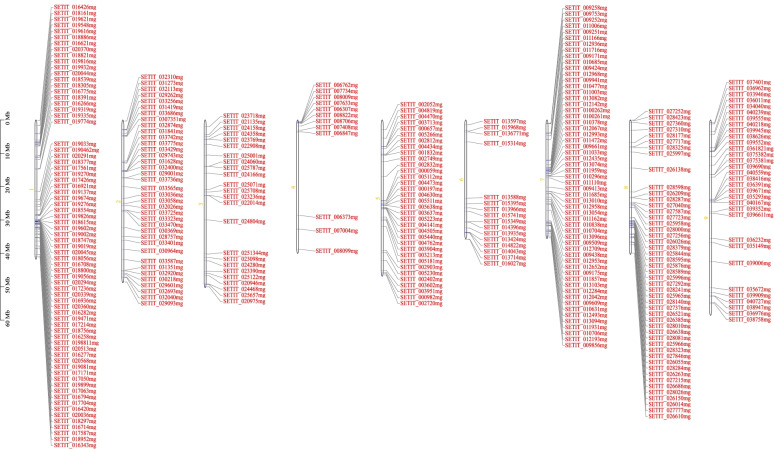
Physical map of differentially expressed genes (DEGs) commonly existed in each comparison showing their chromosomal localization. The scale bar at left indicates chromosome length

Based on their chromosomal location, the 335 common DEGs shared in all comparisons were plotted onto 9 chromosomes of foxtail millet and the physical map was constructed, through which it was found that these genes were distributed unevenly on the 9 chromosomes (Fig. 2). For example, 71 genes were localized at chromosomes 1, while chromosomes 4 and 6 had 12 and 16 genes, respectively. The chromosome location analysis showed important positional information of candidate genes related to tannin content for further development of molecular markers.

### qRT-PCR verification

**Fig. 3:**
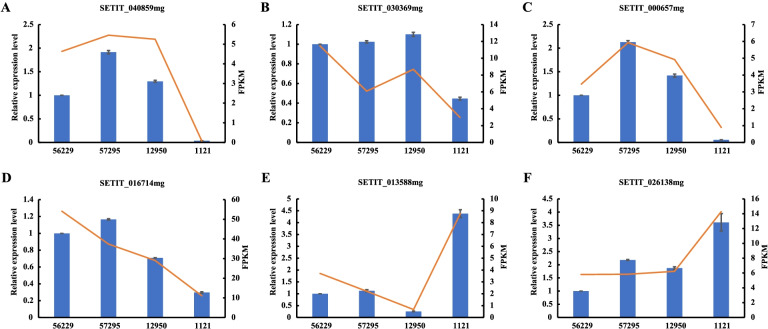
The expression pattern of 6 differentially expressed genes (DEGs) in the leaves of foxtail millet varieties with different levels of leaf tannin content. Transcription levels were verified by qRT-PCR with *SiACTIN* gene as an internal control. The X axis represents different foxtail millet varieties, and the Y axis (left side) indicates the relative expression level of selected genes determined by qRT-PCR (blue columns). The transcript level of genes in variety 56229 was set as 1.0, and error bars represent standard error (*n* = 3). The Y axis (right side) depicts the expression level of genes in RNA-seq data (evaluated by FPKM, red lines)

To verify the reliability of gene changes in transcriptome analysis, 6 DEGs were randomly selected to analyze their transcriptional levels by qRT-PCR. As shown in Fig. 3, the alteration pattern of these genes was consistent with that of transcriptome analysis, indicating that the DEGs identified by comparative transcriptome analysis were reliable. Among these genes, SETIT_040859mg, SETIT_030369mg, SETIT_000657mg and SETIT_016714mg, were significantly up-regulated, whereas SETIT_013588mg and SETIT_026138mg were remarkably down-regulated in foxtail millet varieties with high leaf tannin content.

### GO functional enrichment of DEGs

**Fig. 4:**
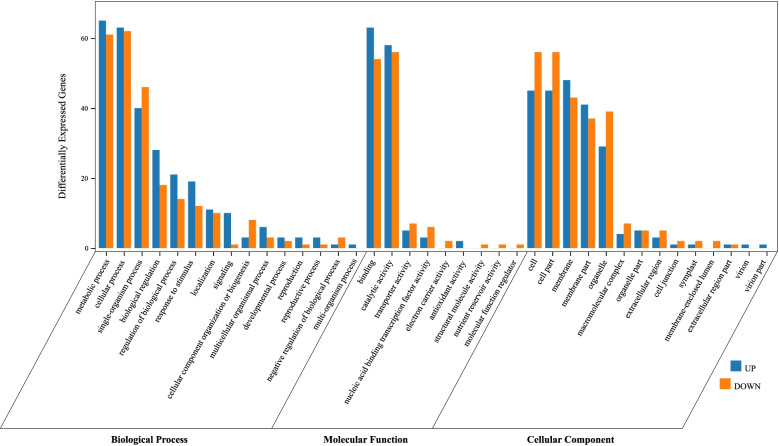
GO enrichment analysis of differentially expressed genes (DEGs). The GO categories include biological processes, molecular functions, cellular components and 38 subcategories. Blue represents up-regulated DEGs in varieties with high leaf tannin content, and red represents down-regulated DEGs

As shown in Fig. 4, the GO annotation results of 335 DEGs among different samples were obtained. These DEGs were classified into 3 categories, including biological process (BP), molecular function (MF), cellular component (CC) and 38 subcategories, in which 39.5%, 23.7% and 36.8% subcategories were classified as BP, MF and CC, respectively (Table S4). In BP terms, metabolic process (GO:0008152), cellular process (GO:0009987) and single-organism process (GO:0044699) showed significant enrichment. In MF terms, binding (GO:0005488) and catalytic activity (GO:0003824) indicated significant enrichment. In CC terms, cell (GO:0005623), cell part (GO:0044464), membrane (GO:0016020), membrane part (GO:0044425) and organelle (GO:0043226) had more numbers of genes. These results indicated that these biological processes, molecular functions and cellular components had undergone remarkable changes in foxtail millet varieties with different levels of tannin content.

### KEGG pathway enrichment analysis of DEGs

**Fig. 5:**
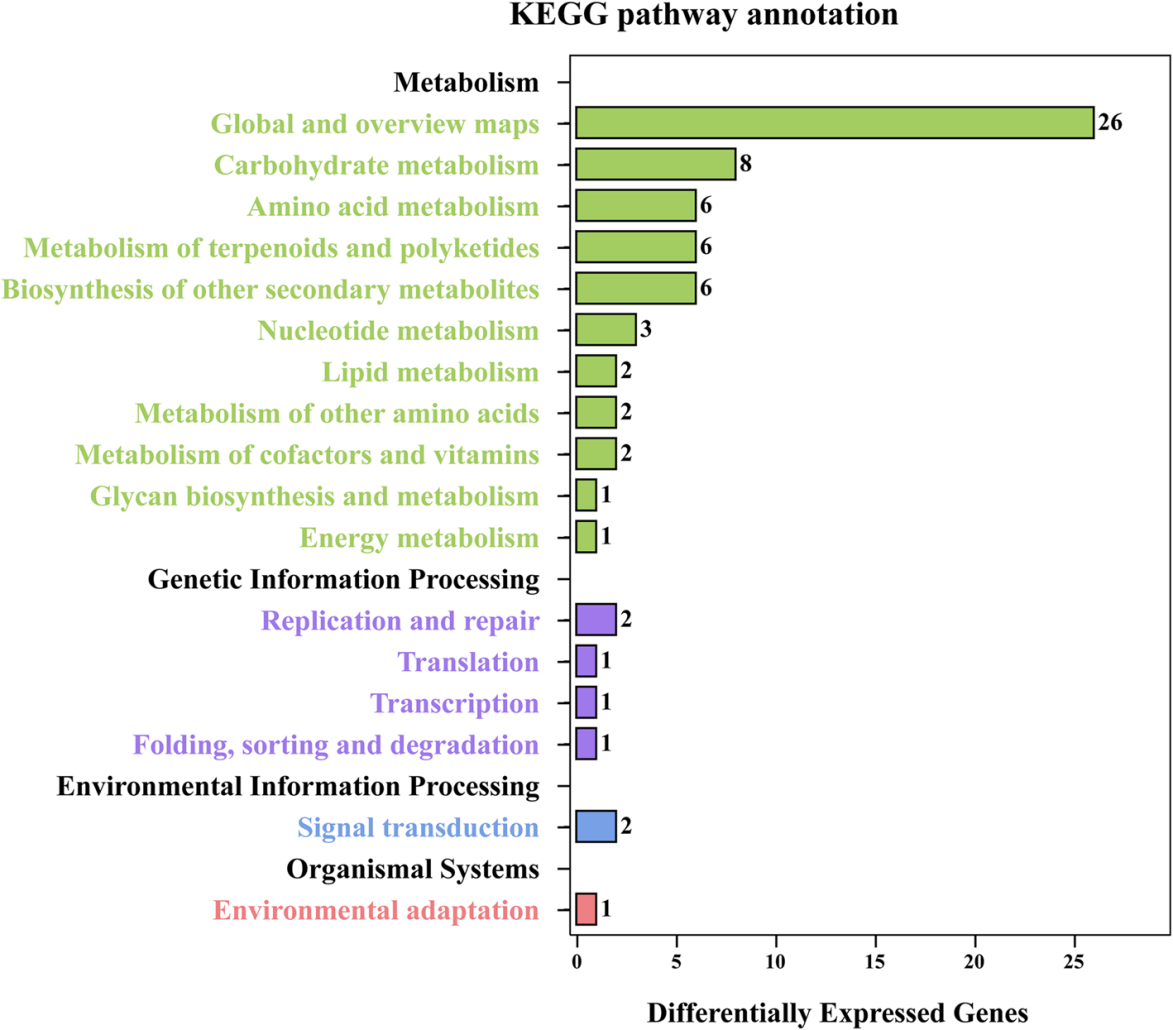
KEGG classification of differentially expressed genes (DEGs). The name of KEGG pathway is shown on the Y axis, and the number of DEGs annotated to the pathway is shown on the X axis

To further identify the biological pathways and their function in the leaf tissues of foxtail millet, the 335 DEGs were subjected to KEGG pathway analysis. A total of 115 DEGs were assigned to 50 KEGG pathways, among which 26 DEGs (22.61%) were involved in metabolic pathways, similarly, 17 DEGs (14.78%) for biosynthesis of secondary metabolites, and 4 DEGs (3.48%) of phenylpropanoid biosynthesis (Table S5). The 50 pathways belong to 4 main categories that could be further divided into 17 subcategories. As shown in Fig. 5, the metabolism category had the highest proportion, followed by genetic information processing, environmental information processing and organismal systems.

Precursor molecules for tannins biosynthesis were reported to be derived from the phenylpropanoid pathway [30]. As per the KEGG analysis, the genes SETIT_009509mg and SETIT_029093mg present in phenylalanine/tyrosine ammonia-lyase (PTAL) [EC:4.3.1.25], SETIT_014043mg present in peroxidase [EC1.11.1.7] and SETIT_006847mg present in cinnamoyl-CoA reductase (CCR) [EC:1.2.1.44] were enriched into the phenylpropanoid biosynthesis pathway (Figure S2). Except for the gene SETIT_029093mg, they all had higher expression in leaf tissues of foxtail millet varieties with high tannin content as compared to gene expression in varieties with low tannin content.

### Transcription factor prediction

**Fig. 6:**
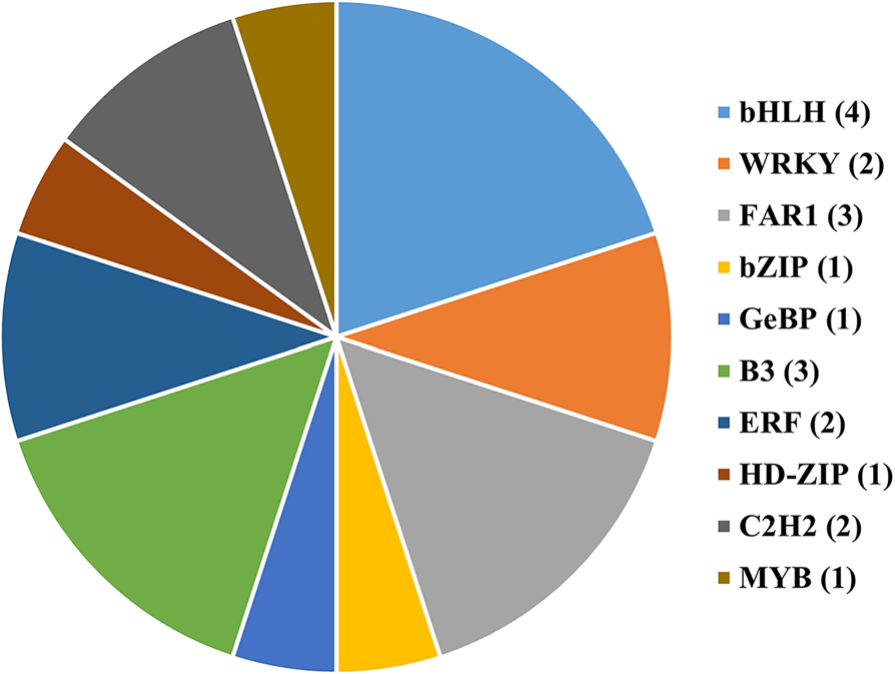
Transcription factor families enriched in differentially expressed genes (DEGs). The number of members in each transcription factor family is presented within the brackets

The GO annotation indicated that regulation of transcription (GO:0006355) showed significant enrichment in biological process, implying that several DEGs may function as transcription factors (TFs) to involve in gene control and regulation of tannins biosynthesis in foxtail millet. Therefore, the potential TF genes were identified among the 335 common DEGs according to the family assignment rules illustrated in PlantTFDB [29]. A total of 20 DEGs were predicted to be involved in the regulation of transcription and were classified into 10 TF families (Fig. 6, Table S6). The most abundant TF families enriched were bHLH followed by FAR1 and B3. Among these TF genes, 13 were up-regulated and 7 were down-regulated.

## Discussion

During growth and maturation period in plants, the secondary metabolites have essential roles. As one of the most important secondary metabolites, tannins is a diverse group of compounds that are related primarily in their ability to complex with proteins [31, 32], and is closely associated with plant defense mechanisms against insect. In this study, 335 DEGs were identified through Illumina sequencing among varieties with different levels of tannin content, which may be in relation to tannins biosynthesis in foxtail millet. Our findings provide valuable resources for investigation on the biosynthesis and regulation mechanisms of tannins in foxtail millet, and for development of molecular markers aimed at marker-assisted selection (MAS) for breeding of varieties with low tannin content, which could be used for interplanting with Chinese chestnut trees to confer protection against red spider attack.

According to their chemical structure and properties, tannins are divided into 2 main groups, condensed tannins (CTs) and hydrolysable tannins (HTs), and CTs are usually found higher concentrations in plants than HTs [33]. CTs, also known as proanthocyanidins (PAs), are synthesized via the phenylpropanoid and flavonoid pathways, and are widespread in the plant kingdom [30, 34]. In the present study, we found several genes were enriched into flavonoid and phenylpropanoid pathways according to the gene analyses. For example, the DEGs, SETIT_009941mg, SETIT_004762mg, SETIT_033256mg and SETIT_006373mg, were annotated to flavonoid biosynthetic process (GO:0009813), and SETIT_017426mg was annotated to flavonoid metabolic process (GO:0009812). As per the KEGG enrichment analysis, the 2 DEGs, SETIT_009509mg and SETIT_029093mg, which present in phenylalanine/tyrosine ammonia-lyase (PTAL) [EC:4.3.1.25], may code for enzymes in the early steps of the phenylpropanoid pathway [30, 35], and significantly affect the CTs biosynthesis and levels in foxtail millet. Our results suggested that these DEGs may be key genes involved in biosynthesis of tannins in foxtail millet, and be utilized for MAS breeding program and generating low-tannin content varieties. However, their functions still need to be further clarified.

The biosynthesis pathway of CTs is under complex control by multiple regulatory genes at the transcriptional level [36]. According to gene structure, these regulatory genes are classified into 6 different families, including bHLH, MYB, WD40, WRKY, MADS and WIP transcription factors, and most of the regulatory elements belong to the top 3 families. For example, the gene *TRANSPARENT TESTA8* (*VfTT8*), encoding a bHLH transcription factor, was identified as the candidate for *zt2*, a gene controls absence of tannin in faba bean [37]. In tetraploid cotton, 2 R2R3-type MYB transcription factors, GhMYB36 and GhMYB10, were identified to involve in regulating CTs biosynthesis [38]. A WRKY transcription factor, McWRKY71, was identified in *Malus* crabapple, and overexpression of *McWRKY71* promotes the accumulation of CTs in apple calli. Besides, it was found that McWRKY71 directly binds to the W-BOX element in the promoter of *McANR*, which promotes CTs accumulation, and McWRKY71 interacts with McMYB12, a functional regulator of CTs synthesis [39]. In this study, 20 genes were predicted to encode transcription factors categorized into 10 families, including 4 bHLH members, 1 MYB member and 2 WRKY members, and they were differentially expressed in foxtail millet varieties with different levels of tannin content. Therefore, these genes may be key candidates for involving in regulation of biosynthesis and accumulation of tannins in foxtail millet, and their function and regulation mechanism will be investigated in future work.

It is well known that CTs accumulate in the vacuole, while the known enzymes participate in the biosynthesis pathway mostly locate on the endoplasmic reticulum membranes or in the cytoplasm [40]. Hence, the CTs precursors must be transported from the site of synthesis to the site of storage by 2 models: the vesicle trafficking-mediated model and the membrane transporter-mediated model [41, 42]. The latter is established based on 3 types of transporters, glutathione S-transferase (GST), multidrug and toxic compound extrusion (MATE) and H^+^-ATPase. For example, a GST gene family member in *Arabidopsis thaliana*, TT19, was reported to localize both in the cytoplasm and on the tonoplast, and is required for transferring the anthocyanins into the vacuole [43]. In the current study, based on the GO enrichment analysis, we found a DEG, SETIT_009171mg, was annotated to transmembrane transport process (GO:0055085) and ATPase activity, coupled to transmembrane movement of substances (GO:0042626). Furthermore, the DEG SETIT_002832mg was categorized to glutathione metabolic process (GO:0006749) and glutathione transferase activity (GO:0004364). As per the KEGG analysis, this gene was present in glutathione S-transferase [EC:2.5.1.18]. Whether these genes involve in transport processes required for CTs biosynthesis is also an interesting focus of our future work.

## Conclusion

In this study, by using RNA-seq data derived from 4 foxtail millet varieties with different levels of tannin content, we identified 335 differentially expressed genes, which were potential candidate genes contributed to tannins biosynthesis in foxtail millet. To understand their functions and biological pathways they involved in, GO and KEGG enrichment analyses were performed. Moreover, the transcription factors involved in the regulation of tannins biosynthesis pathway were predicted by using the PlantTFDB database. These results will provide a basis for investigation on biosynthesis and regulation mechanisms of tannins in foxtail millet, and provide a resource for breeding of low-tannin varieties through marker-assisted selection, which could be utilized for interplanting with Chinese chestnut tree and prevent red spider attack.

## Supplementary Information

**Additional file 1:** Supplementary file **Table S1.** The tannin content in leaves of different foxtail millet varieties. **TableS2.** Primer sequences used for quantitative real-time PCR. **Table S3.** Characteristics of the RNA-sequencing data obtained from analysis of 4 leaf samples of foxtail millet. **Table S4.** GO annotation of the differentially expressed genes shared in all comparisons. **Table S5.** KEGG pathway annotation and classification of the differentially expressed genes shared in all comparisons. **Table S6.** The transcription factor (TF) genes predicted using PlantTFDB. Up and down indicate gene is up-regulated and down-regulated in 3 foxtail millet varieties with high leaf tannin content, respectively. **Figure S1.** Heatmap of pairwise Pearson correlation coefficients (R^2^) between samples. **Figure S2.** KEGG analysis of phenylpropanoid pathway in foxtail millet. The pathway map image is obtained from KEGG [1-3].

## Data Availability

The datasets generated and/or analyzed during the current study are available in the Sequence Read Archive (SRA) of the National Center for Biotechnology Information (NCBI) under the accession number PRJNA772942.

## Abbreviations

RAPD: Random Amplified Polymorphic DNA
SCAR: Sequence-Characterised Amplified Region
SNP: Single Nucleotide Polymorphism
SSR: Simple Sequence Repeat
KASP: Kompetitive Allele-Specific PCR
FPKM: Fragments per kilobase of transcript per million mapped reads
DEG: Differentially expressed gene
qRT-PCR: Quantitative real-time PCR
GO: Gene Ontology
KEGG: Kyoto Encyclopedia of Genes and Genomes
BP: Biological process
MF: Molecular function
CC: Cellular component
PTAL: Phenylalanine/tyrosine ammonia-lyase
CCR: Cinnamoyl-CoA reductase
TF: Transcription factor
MAS: Marker-assisted selection
CTs: Condensed tannins
HTs: Hydrolysable tannins
TT8: TRANSPARENT TESTA8
GST: Glutathione S-transferase
MATE: Multidrug and toxic compound extrusion
