# Supplementary material for: Transcriptome analysis and characterization of genes associated to leaf tannin content in foxtail millet [Setaria italica (L.) P. Beauv.]
Source: BMC Genomics. 2022 Jul 14;23:512. doi: 10.1186/s12864-022-08746-8 (PMC9284691; doi:10.1186/s12864-022-08746-8)
Supplement: Supplementary file 1 — Additional file 1: Supplementary file Table S1. The tannin content in leaves of different foxtail millet varieties. TableS2. Primer sequences used for quantitative real-time PCR. Table S3. Characteristics of the RNA-sequencing data obtained from analysis of 4 leaf samples of foxtail millet. Table S4. GO annotation of the differentially expressed genes shared in all comparisons. Table S5. KEGG pathway annotation and classification of the differentially expressed genes shared in all comparisons. Table S6. The transcription factor (TF) genes predicted using PlantTFDB. Up and down indicate gene is up-regulated and down-regulated in 3 foxtail millet varieties with high leaf tannin content, respectively. Figure S1. Heatmap of pairwise Pearson correlation coefficients (R2) between samples. Figure S2. KEGG analysis of phenylpropanoid pathway in foxtail millet. The pathway map image is obtained from KEGG [1-3]. [file 12864_2022_8746_MOESM1_ESM.zip › Supplementary file/Supplementary file.docx]

Supplementary file

**Table S1.** The tannin content in leaves of different foxtail millet varieties.

| Variety | tannin content of the leaves (%) |
| --- | --- |
| 57295 | 0.292 |
| 56229 | 0.280 |
| 12950 | 0.276 |
| 1121 | 0.232 |
| JG32 | 0.283 |
| HK950 | 0.226 |

**Table S2.** Primer sequences used for quantitative real-time PCR.

| Gene name | Primer sequence (5’-3’) | |
| --- | --- | --- |
|  | Forward | Reverse |
| SETIT_040859mg | TGGAGACAGTGGAGAGATCGATT | TCCTCAGGTTGAGCAGTGACA |
| SETIT_030369mg | GATACACCCGCCGTTGCT | CTCCCATGATGAACACGGTCTT |
| SETIT_000657mg | CGTCCCAGCTATGCGTCAT | TCCGGAACGAGCCCAAA |
| SETIT_016714mg | GGAAGGTGGACAAGACGTTAGC | TGCTTTTGGTGCCTTTTGC |
| SETIT_013588mg | TGAGTACCGTATGGCTCATTTCA | ACCAGCTCATCAGAAACATGGA |
| SETIT_026138mg | ATTCTCGGCTCCGCTTCCT | ACACGGACACCGTCATTCG |
| SiACTIN | GGATACTCTTTCACCACCTC | ACCTCAGGGCACCTAAAC |

**Table S3.** Characteristics of the RNA-sequencing data obtained from analysis of 4 leaf samples of foxtail millet.

| Sample name | Raw reads (M) | Clean reads (M) | Percent (%) | Clean bases (G) | Mapped reads (%) |
| --- | --- | --- | --- | --- | --- |
| 57295-1 | 41.444 | 40.187 | 96.97 | 12.023 | 95.39 |
| 57295-2 | 39. 790 | 39.688 | 99.74 | 11.865 | 95.48 |
| 57295-3 | 52.574 | 52.112 | 99.12 | 15.523 | 95.65 |
| 56229-1 | 37.857 | 37.768 | 99.76 | 11.257 | 95.75 |
| 56229-2 | 43.064 | 42.930 | 99.69 | 12.841 | 95.49 |
| 56229-3 | 44.932 | 44.766 | 99.63 | 13.378 | 95.30 |
| 12950-1 | 38.042 | 37.692 | 99.08 | 11.261 | 94.76 |
| 12950-2 | 47.110 | 46.880 | 99.51 | 13.999 | 95.28 |
| 12950-3 | 44.196 | 43.962 | 99.47 | 13.134 | 95.27 |
| 1121-1 | 41.028 | 40.871 | 99.62 | 12.224 | 94.85 |
| 1121-2 | 50.023 | 49.902 | 99.76 | 14.918 | 94.94 |
| 1121-3 | 38.949 | 38.839 | 99.72 | 11.622 | 94.93 |

The suffixes -1, -2, -3 indicate three biological replicates for each sample.

**Table S4.** GO annotation of the differentially expressed genes shared in all comparisons.

**Table S5.** KEGG pathway annotation and classification of the differentially expressed genes shared in all comparisons.

**Table S6.** The transcription factor (TF) genes predicted using PlantTFDB. Up and down indicate gene is up-regulated and down-regulated in 3 foxtail millet varieties with high leaf tannin content, respectively.

| No. | TF family | Gene numbers | Gene ID | Change of expression level |
| --- | --- | --- | --- | --- |
| 1 | bHLH | 4 | SETIT_002720mg | down |
|  |  |  | SETIT_010685mg | up |
|  |  |  | SETIT_015349mg | up |
|  |  |  | SETIT_017063mg | up |
| 2 | WRKY | 2 | SETIT_010706mg | down |
|  |  |  | SETIT_027360mg | up |
| 3 | FAR1 | 3 | SETIT_015741mg | down |
|  |  |  | SETIT_019816mg | up |
|  |  |  | SETIT_028177mg | up |
| 4 | bZIP | 1 | SETIT_018161mg | down |
| 5 | GeBP | 1 | SETIT_020045mg | up |
| 6 | B3 | 3 | SETIT_0100261mg | up |
|  |  |  | SETIT_0100262mg | up |
|  |  |  | SETIT_010378mg | up |
| 7 | ERF | 2 | SETIT_002749mg | up |
|  |  |  | SETIT_019270mg | down |
| 8 | HD-ZIP | 1 | SETIT_018305mg | down |
| 9 | C2H2 | 2 | SETIT_019899mg | down |
|  |  |  | SETIT_035672mg | up |
| 10 | MYB | 1 | SETIT_017704mg | up |

**Figure S1.** Heatmap of pairwise Pearson correlation coefficients (R^2^) between samples.

**
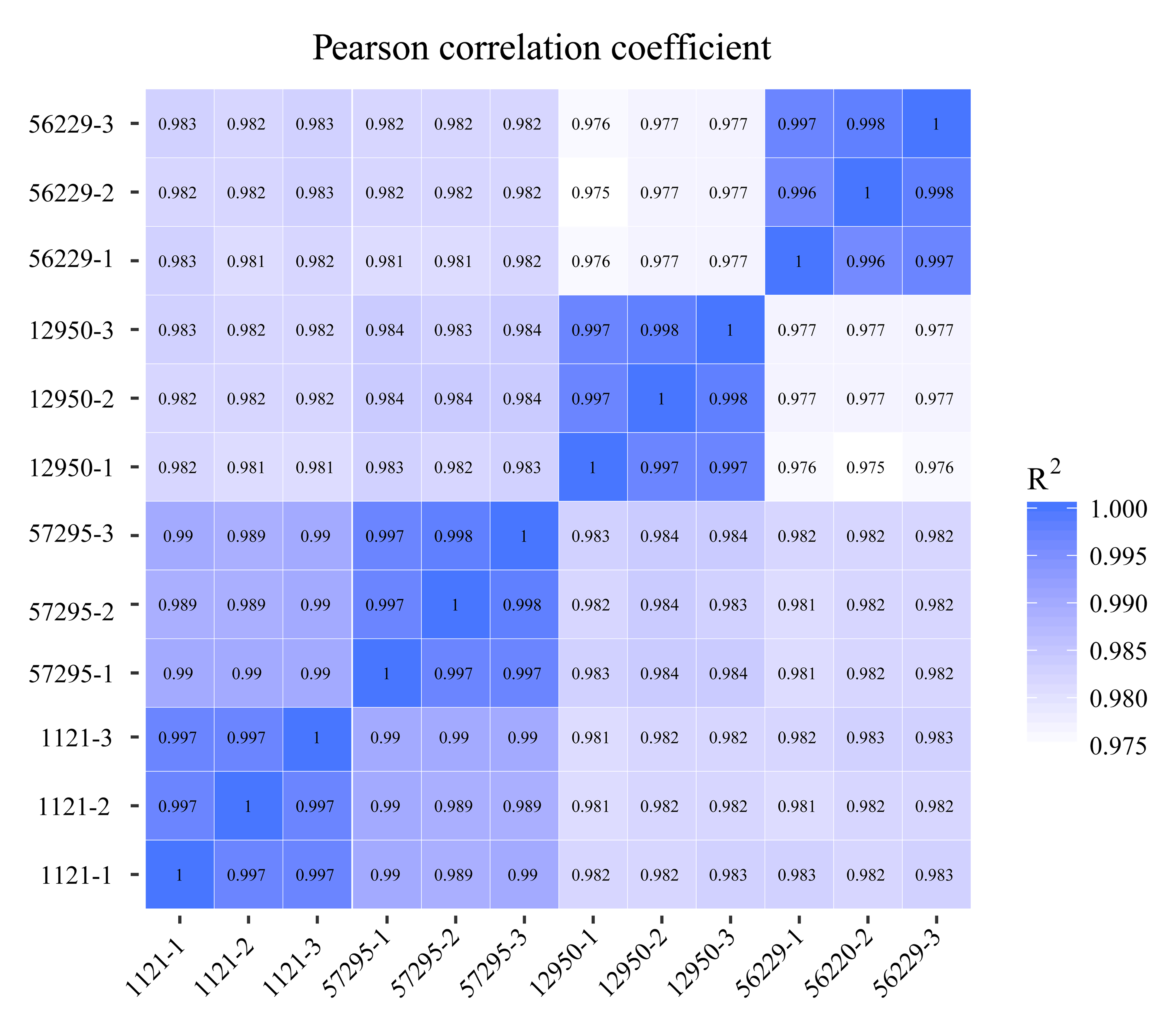
**

**Figure S2.** KEGG analysis of phenylpropanoid pathway in foxtail millet. The pathway map image is obtained from KEGG [1-3].


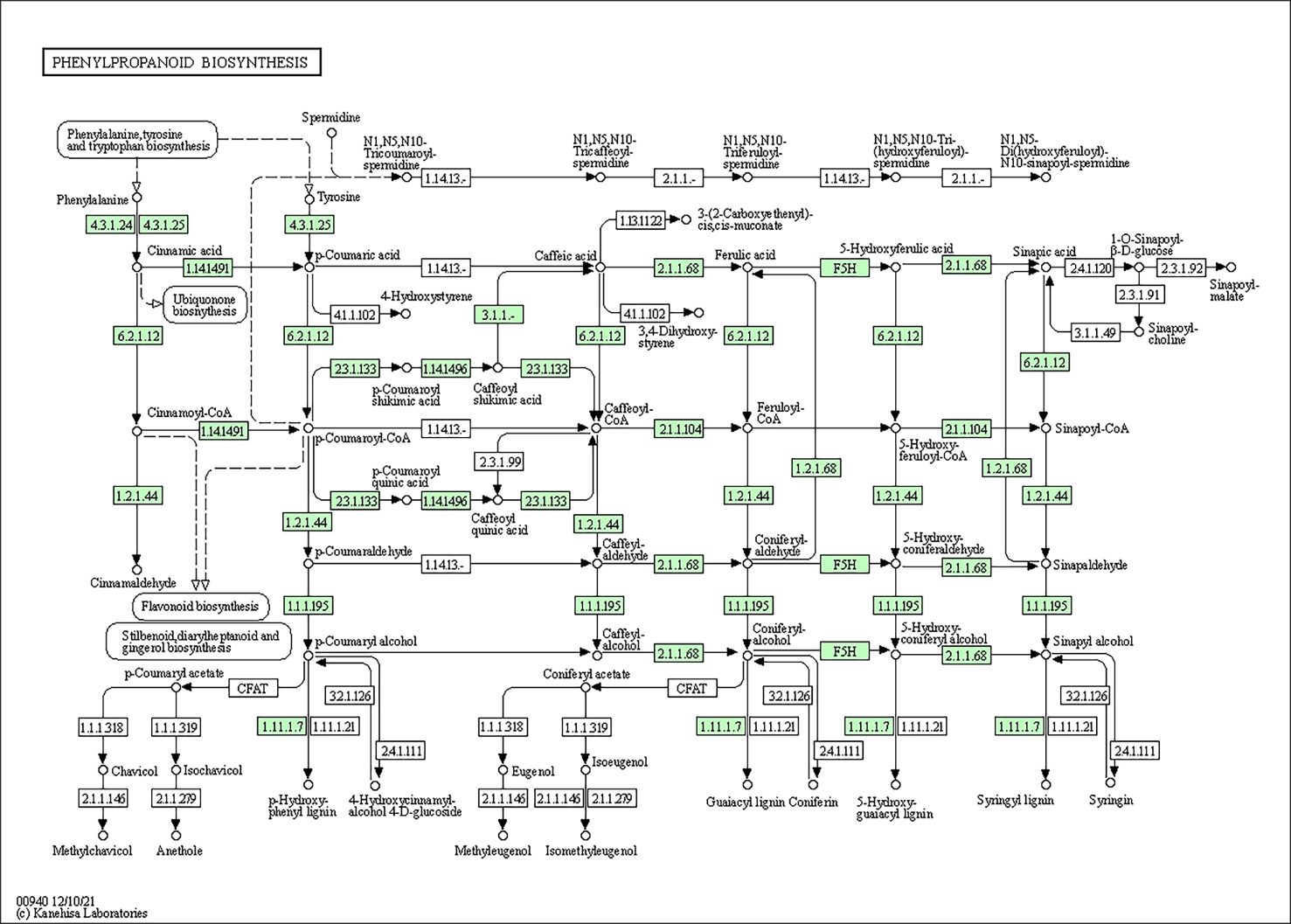


References

1. Kanehisa M, Goto S. KEGG: Kyoto encyclopedia of genes and genomes. Nucleic Acids Res. 2000; 28(1):27-30.

2. Kanehisa M, Furumichi M, Sato Y, Ishiguro-Watanabe M, Tanabe M. KEGG: integrating viruses and cellular organisms. Nucleic Acids Res. 2021; 49(D1):D545-D551.

3. Kanehisa M. Toward understanding the origin and evolution of cellular organisms. Protein Sci. 2019; 28(11):1947-1951.
